# Dogs Evaluate Threatening Facial Expressions by Their Biological Validity – Evidence from Gazing Patterns

**DOI:** 10.1371/journal.pone.0143047

**Published:** 2016-01-13

**Authors:** Sanni Somppi, Heini Törnqvist, Miiamaaria V. Kujala, Laura Hänninen, Christina M. Krause, Outi Vainio

**Affiliations:** 1 Department of Equine and Small Animal Medicine, Faculty of Veterinary Medicine, University of Helsinki, Helsinki, Finland; 2 Cognitive Science, Faculty of Behavioural Sciences, University of Helsinki, Helsinki, Finland; 3 Department of Neuroscience and Biomedical Engineering, Aalto University, Espoo, Finland; 4 Department of Production Animal Medicine, Faculty of Veterinary Medicine, University of Helsinki, Helsinki, Finland; University of Lincoln, UNITED KINGDOM

## Abstract

Appropriate response to companions’ emotional signals is important for all social creatures. The emotional expressions of humans and non-human animals have analogies in their form and function, suggesting shared evolutionary roots, but very little is known about how animals other than primates view and process facial expressions. In primates, threat-related facial expressions evoke exceptional viewing patterns compared with neutral or positive stimuli. Here, we explore if domestic dogs (*Canis familiaris*) have such an attentional bias toward threatening social stimuli and whether observed emotional expressions affect dogs’ gaze fixation distribution among the facial features (eyes, midface and mouth). We recorded the voluntary eye gaze of 31 domestic dogs during viewing of facial photographs of humans and dogs with three emotional expressions (threatening, pleasant and neutral). We found that dogs’ gaze fixations spread systematically among facial features. The distribution of fixations was altered by the seen expression, but eyes were the most probable targets of the first fixations and gathered longer looking durations than mouth regardless of the viewed expression. The examination of the inner facial features as a whole revealed more pronounced scanning differences among expressions. This suggests that dogs do not base their perception of facial expressions on the viewing of single structures, but the interpretation of the composition formed by eyes, midface and mouth. Dogs evaluated social threat rapidly and this evaluation led to attentional bias, which was dependent on the depicted species: threatening conspecifics’ faces evoked heightened attention but threatening human faces instead an avoidance response. We propose that threatening signals carrying differential biological validity are processed via distinctive neurocognitive pathways. Both of these mechanisms may have an adaptive significance for domestic dogs. The findings provide a novel perspective on understanding the processing of emotional expressions and sensitivity to social threat in non-primates.

## Introduction

Appropriate reading and responding to companions’ emotional signals is important to social creatures. For humans, facial expressions are the main channel for communicating emotional states and have thus been at the center of scientific interest for decades [1,2]. Most mammalian species have musculature for producing facial movements that resemble human expressions [3,4]. The analogies in the form and function of human and non-human animal emotional expressions suggest shared evolutionary roots [2,4,5]. Nevertheless, little is known about how non-primates view and process facial expressions. Full understanding of the complexity and cognitive mechanisms of facial expressions requires a wider approach, taking into account various examples from the animal kingdom (for reviews, see [4,6,7]).

Humans, chimpanzees (*Pan troglodytes)* and macaques (*Macaca mulatta*) exhibit exceptional viewing behavior toward threat-related expressions compared with neutral or positive stimuli. Typically the threatening stimuli accelerate detection [8,9] or prolong attention [9–16], but sometimes they evoke aversive reactions [9,17–19]. The phenomenon has been extensively studied because it is particularly evident in anxiety and mood disorders [20–22]. The attentional bias toward threat is considered to originate from a phylogenetically old adaptive mechanism: the sensitivity to detect and avoid threats represents a survival advantage [21]. Studying sensitivity to social threat in animals other than humans can promote understanding of the origins of phenomenon [15].

During viewing of emotional faces different facial features attract attention differently [23,24]. In humans and other primates the viewed emotional expression alter the distribution of gaze fixations among inner key structures, i.e. eyes, nose and mouth [10,25–31], probably because these features convey different emotional information for a viewer [24,27–29]. It is not known how other animals than primates scan facial expressions and what facial features are relevant for them.

Here, we chose domestic dogs (*Canis familiaris*) as study subjects because canids use facial cues richly in social interactions and their facial expressions share similarities with primates [5,32,33]. In addition, domestic dogs are known for their well-developed social communication skills (for reviews, see [34,35]) and they respond flexibly to human emotional signals [36–39]. The facial expressions of dogs can be linked to affective situations (e.g. anticipation of the reward, social isolation or reunion with the owner) [40–43] and categorized according to basic emotion classification used for humans [41]. Certain characteristics of facial communication may have contributed to the relation between dogs and humans, and even given a selective advantage to dogs during domestication [43]. However, only scarce information exists considering how dogs process facial expressions of conspecifics’ and humans. To date we know that dogs can distinguish human facial expressions from images after discrimination training [44,45] and their viewing behavior is sensitive to the facial expressions of both conspecifics and humans [46].

The approaches in which human and non-human emotional perception have been studied have differed greatly both in methodological constraints and theoretical aspects [4]. In humans and other primates, eye gaze tracking has been a widely used method for assessing affective responses. To narrow the methodological gap between the studies of primates and non-primates, we employed the rapidly advanced eye tracking methodology [47–50] to measure the voluntary eye gaze of domestic dogs during viewing of emotional expressions of dogs and humans. Eye tracking findings to date show that the gazing behavior of dogs has analogies with that of primates: dogs spontaneously focus attention on informative objects, like faces [49] and eyes [50]; they prefer images representing conspecifics over images of human or inanimate objects [49], upright faces over inverted ones [50] and familiar faces over strange ones [50]. While watching dynamic social stimuli, dogs gazing behavior also resembles their behavior in real communicative situations [48]. Together these findings suggest that dogs comprehend two-dimensional representations and their gaze targets are altered by the seen contextual information.

The aim of the present study was to explore how dogs scan facial expressions of conspecifics (social and phylogenetic stimuli) compared with the species that is most likely emotionally relevant for them, humans (social, but non-phylogenetic stimuli). We were particularly interested to discover whether dogs exhibit specific attentional biases toward potentially threatening social stimuli versus non-threatening social stimuli, and whether their gaze distribution among the inner facial features is dependent on the seen emotional content.

## Materials and Methods

### Ethical statement

This study was performed in strict accordance with the Finnish Act of Animal Experimentation (62/2006) in which the European convention for the protection of vertebrate animals used for experimental and other scientific purposes (Directive 86/609/EEC) is fully implemented. All protocols were approved by the Ethical Committee for the Use of Animals in Experiments at the University of Helsinki (2/2010) and the Finnish national Animal Experiment Board (approval#STH367A/ESLH-2008-04236/Ym-23). Animals were not harmed in any way during the experiments and neither mechanical nor manual restraint was applied. Only non-invasive and reward-based methods were used during training and measurement.

### Animals and pre-training

All experiments were conducted at the Veterinary Faculty of the University of Helsinki. Participant dogs (25 privately owned pet dogs and 8 kennel-housed beagles) were on average 4.6 years old (SD 2.2), and represented 13 different breeds and mongrels (8 beagles, 6 border collies, 3 hovawarts, 3 beauce shepherds, 2 rough collies, 2 smooth collies, 1 great Pyrenees, 1 Welsh corgi, 1 Australian kelpie, 1 lagotto romagnolo, 1 Manchester terrier, 1 Swedish vallhund, 1 Finnish lapphund, 2 mongrels). The daily routines of the dogs were kept similar to those in their regular life. Pet dogs (14 intact females, 4 sterilized females, 5 intact males and 2 castrated males) lived in their home environment, were fed once or twice a day and taken outdoors three to five times for 0.5–2 hours at a time. Kennel dogs (2 sterilized females and 6 castrated males) lived in the kennel facilities of the University of Helsinki. They were fed twice a day and released into an exercise enclosure once a day for two hours.

All the dogs had previously participated in an eye-tracking experiment [50] but were naïve regarding images used in the present study. Prior the previous eye-tracking experiment, all subjects were clicker-trained to lie still in front of a monitor and lean their jaw on a specially designed chin rest [49]. The criterion for passing the training period was that the dog took the pre-trained position without being commanded to do so and remained in that position for at least 30 seconds while the owner and experimenters were positioned behind an opaque barrier. During the training, the dogs were not encouraged to fix their eyes on a monitor or images and they were not restrained or forced to perform the task.

### Stimuli

**Fig 1:**
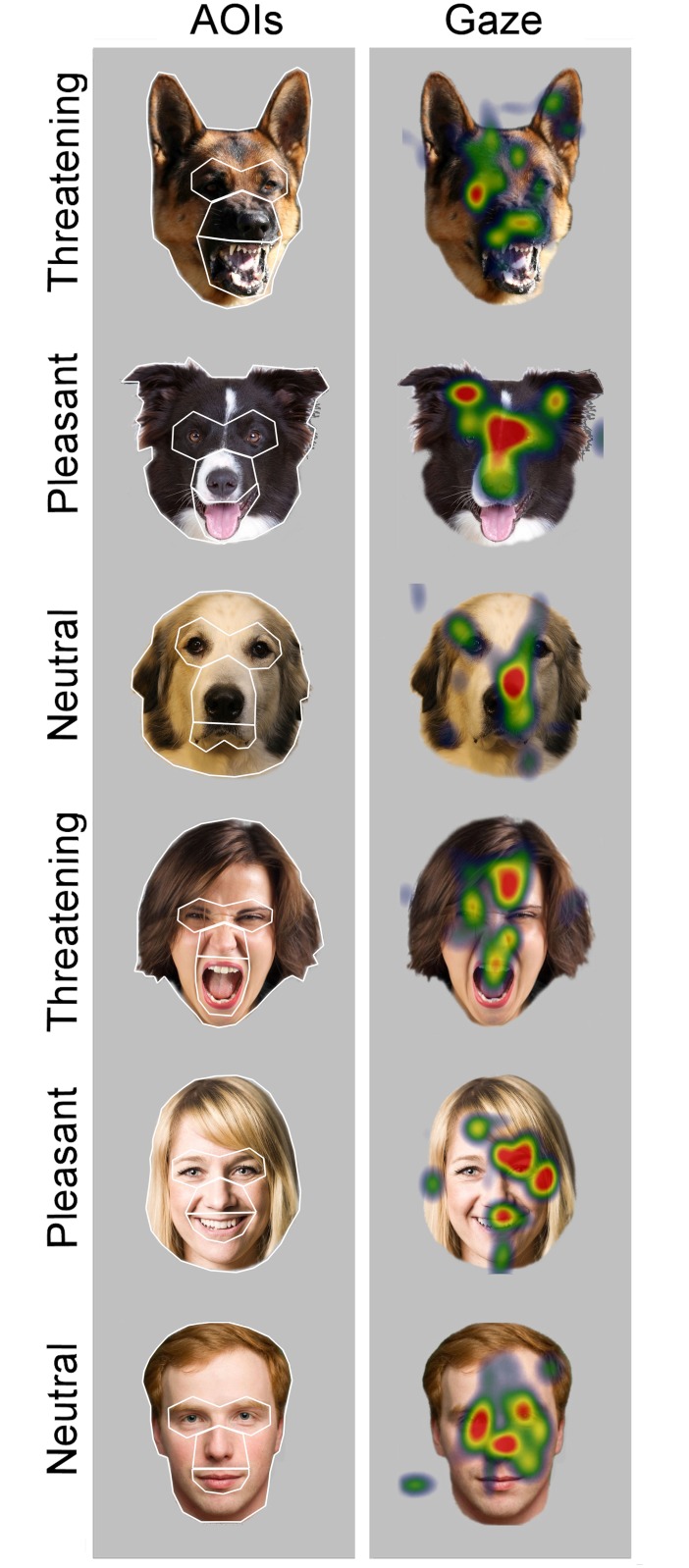
Examples of stimulus images, areas of interest and the gaze patterns of dogs. The gaze data were collected from five areas of interest (AOIs) of three facial expressions (Threatening, Pleasant and Neutral) of dogs and humans. The AOIs are drawn in the left panel: eyes, midface, mouth, inner face (eyes, midface and mouth combined) and the whole face. In the right panel, the averaged gaze fixation distributions of 12 dogs during 1500 ms presentations are given with color coding: the minimum fixation duration (≤ 5 ms) is indicated by light blue and maximum (≥ 100 ms) by bright red. See also S2 Movie for examples of scanning patterns. The original photos by S. Somppi and 123rf^®^. The images were purchased with a license to publish these images in electronic and printed documents and reports.

Digital color photographs of unfamiliar dog and human faces with direct gaze and three emotional expressions were used as stimulus images (Fig 1). Expressions were Threatening (10 aggressive dog faces; 10 angry human faces), Pleasant (10 positively valenced dog faces; 10 smiling human faces) and Neutral (10 expressionless dogs; 10 expressionless humans) (Table 1). The dog faces represented a total of 25 different breeds. Half of the human faces were images of women, half of men, balanced over expressions. Human portraits were acquired from royalty-free online stocks (e.g. MS ClipArt, 123RF^®^, BigStock). Canine photographs were acquired from authors’ and photographer Aino Pikkusaari’s collections, online image services (Google, Wikimedia Commons, Flickr) and royalty-free online stocks (123RF^®^, BigStock).

**Table 1:** Facial actions of Dog and Human images with three emotional expressions.

	Dog faces	DogFACS^1^	Human faces	FACS^2^
Threatening	Brows lowered and drawn together; lips parted^3^; upper lip raised; nose wrinkled; tongue shown^3^; ears forward, flattened or rotated backwards^4^	AU4; AU25, AU109; AU110; AU19^3^; EAD101, EAD103 or EAD104^4^	Brows lowered and drawn together; upper eyelids raised; nasolabial deepened; lips parted^3^; upper lip raised; mouth stretched; tongue shown^3^	AU4; AU5; AU11; AU25; AU10; AU27; AU19^3^
Pleasant	Lip corners pulled upwards; lips parted; tongue shown; ears forward	AU12; AU25; AU19; EAD101	Cheeks raised; lip corners pulled upwards; lips parted;	AU6; AU12; AU25
Neutral	Eyes, brows and cheeks relaxed; ears forward	AU0; EAD	Eyes, brows and cheeks relaxed	AU0

^1^ DogFACS applied from [43]. AU4 and AU19 are proposals; they were not documented in [43].

^2^ Human facial action coding system [69].

^3^ Teeth visible; five of the models were teeth together and five were teeth parted with tongue shown.

^4^ Three of the models were ears forward; three ears backward and four ears sideward.

The low-level image properties (luminance, kurtosis, skewness, standard deviation and root mean square contrast) were assessed using MatLab 7.12 (MathWorksInc, USA). In addition, arousal and valence of the stimuli were independently ranked afterwards by 22 adult humans (with varying expertise and experience about dogs), naïve regarding the study protocol (11 females, 11 males, aged 25–46). Mean valence ratings with 7-point scales (1 = negative, 4 = neutral, 7 = positive) were as follows (mean ± SD): Threatening dog 1.79 ± 0.5, Threatening human2.47 ± 0.6, Pleasant dog 4.99 ± 0.6, Pleasant human 5.57 ± 0.4, Neutral dog 3.82 ± 0.5, Neutral human 3.61 ± 0.2. Arousal ratings (1 = not arousing, 7 = highly arousing) for each stimulus category were the following: Threatening dog 5.65 ± 0.6, Threatening human 5.02 ± 0.7, Pleasant dog 3.25 ± 0.9, Pleasant human 4.25 ± 0.7, Neutral dog 2.78 ± 1.0, Neutral human 2.48 ± 0.8.

### Equipment

The binocular eye movements of dogs were recorded with an infrared-based contact-free eye-tracker (iView X^™^ RED250, SensoMotoric Instruments GmbH, Germany), which was integrated below a 22” LCD monitor placed at an average distance of 0.70 m from the dogs’ eyes (SD 0.03 m, ranging between 0.61 and 0.73 m). The distance was adjusted individually for each dog for optimal detection of the eyes with the eye tracker. The monitor, the eye tracker and the chin rest were placed in a cardboard cabin (*h* = 1.5 m, *w* = 0.9 m, *d* = 0.9 m) with three walls and a roof.

The eye tracker was calibrated for each dog’s eyes using a five-point procedure (for details, see [49,50]. The calibrated area was the whole monitor, equal to a visual field of 40.5° x 24.4° from the average distance of 0.70 m. After a successful calibration, the calibration was saved and the accuracy of the calibration was checked twice. Dogs were free to change their position between the calibration and calibration check runs. The average calibration accuracy was 97% (SD 7%, ranging between 80 and 100%), scored as a proportion of fixated points out of five calibration points within 1° margin. Two of the 25 pet dogs were excluded from the experiment because of inadequate calibration accuracy (under 80%). To maximize the attentiveness and motivation of the dogs, the calibration and image viewing sessions were run on separate days. Illumination and the position of the chin rest, monitor, and eye tracker were kept the same during the calibration and the image viewing, and the accuracy of the central point fixations was re-assessed visually immediately before the image viewing. In our previous experiment, the calibrations of six dogs were tested four to six times, resulting in an average accuracy of 84% [49]. Thus, according to our previous findings, the stored calibration can be used repeatedly during separate days.

### Procedure

The stimulus presentation and the eye gaze tracking were conducted using a similar protocol as described in [50]. Dogs viewed a block of 8–12 images (1500 ms per image with 500 ms inter-stimulus-interval) independently while the experimenters and the owners waited behind the opaque barrier (S1 Movie). The stimulus block was followed by a break when the dog was rewarded; in total, six stimulus blocks were recorded per single measurement session. The length of a stimulus block and the order of the stimuli were randomized in order to prevent anticipatory behavior. Two measurement sessions were carried out on separate testing days. The time delays between the testing days were 3–12 days (average 5.2 days, SD 2.2).

### Data processing

A total of 3,479 stimulus images yielded sufficient gaze data (Threatening dog N = 583; Threatening human N = 575; Pleasant dog N = 574; Pleasant human N = 589; Neutral dog N = 592; Neutral human N = 566; S1 Dataset). A total of 241 images were excluded from analysis because of missing data lasting over 750 ms during a single stimulus presentation (interrupted eye tracking due to technical problems or a dog’s behavior). The raw gaze data were further processed using BeGaze 2.4^™^ software (SensoMotoric Instruments GmbH, Berlin, Germany) for defining of gaze fixations. The fixation was encoded if the minimum gaze duration was 75 ms and the maximum dispersion value D = 250 px [49].

Fixations were collected from five areas of interest (AOIs): eyes, midface (i.e. nose / muzzle), mouth (applied from [25,31]) and inner face (covering the combination of eyes, midface and mouth; applied from [11,18]) and the whole face (Fig 1). As the total viewing times vary for different pictures, and the size of the facial features varies for different depicted species, the looking duration was measured as a fixation score, which was normalized by taking into account both the relative size of the facial feature and the total face viewing duration (applied from [31,51,52]). For example, fixation score for the eye area was normalized by subtracting the relative AOI size (e.g. the size of the eyes divided by the size of the whole face) from the relative fixation duration (e.g. the total fixation time targeted to the eyes divided by the total fixation time targeted to the whole face area). Thus, if the value of fixation score is zero, fixations are distributed randomly. Positive fixation scores indicate that the AOI receives longer looking duration than would be expected according to the relative size of the area, and negative fixation scores indicate the opposite [51].

As a measure of speed of the attention, the entry time (i.e. the duration from stimulus onset to the first fixation targeted at certain AOI, ms) was calculated for all AOIs. In addition, the orientation of initial attention (i.e. the spatial distribution of the first fixations) was measured as the 1^st^ fixation probability (%) (applied from [25,31]), which was calculated by counting the number of images where the first fixations hit the particular AOI (inner face, eyes, midface or mouth) and then dividing this value by the number of images where the first fixations hit the whole face area. Further, this proportion was normalized by subtracting the relative AOI size from it.

### Statistical analysis

The statistical analysis was performed on the stimulus category means (S2 Dataset). Comparisons among expression conditions, depicted species and target facial features were conducted with a repeated measures linear mixed-effects model (MIXED) using a first order autoregressive (AR1) covariance structure. In the final model for analyzing the gazing behavior toward the inner face, the fixed effects were depicted species (dog or human), expression (Threatening, Pleasant or Neutral) and an interaction between depicted species and expression. In the final model for analyzing the fixation distribution among the facial features, the fixed effects were depicted species, expression, target facial feature (eyes, midface or mouth) and an interaction between species and facial feature. Other interactions were omitted as being statistically non-significant. The subject (i.e. the tested dog) and the testing day were included as random effects. The group (kennel dog or pet dog) and the gender of the subject were tested as random effects and omitted as being redundant for the models. In addition, the age of the subject, the calibration accuracy, low-level image properties and in the case of entry times, the actual AOI size and the AOI size in relation to the size of the whole face were tested as covariates, but omitted as being not statistically significant. The normality and homogeneity assumptions of the models were checked with normal probability plots of residuals against fitted values. The natural logarithm transformation was used for variables in which the residuals were not normally distributed and the values were back-converted for reporting. The covariance type of the linear mixed-effects models was selected by using the Akaike Information Criteria. Post hoc analyses with Bonferroni test, based on Student's t statistic, were included in the MIXED procedure. The alpha-level was set at 0.05, and the p-values are reported as adjusted values, considering values p < 0.05 as statistically significant (*) and p < 0.08 be a tendency (#). All the results are reported as estimated means with standard errors or means (Mean ± SD). All the statistical analyses were conducted with SPSS statistics 22.0 (IBM, New York, USA).

## Results

### Gazing behavior toward inner face area

#### Fixation score

**Fig 2:**
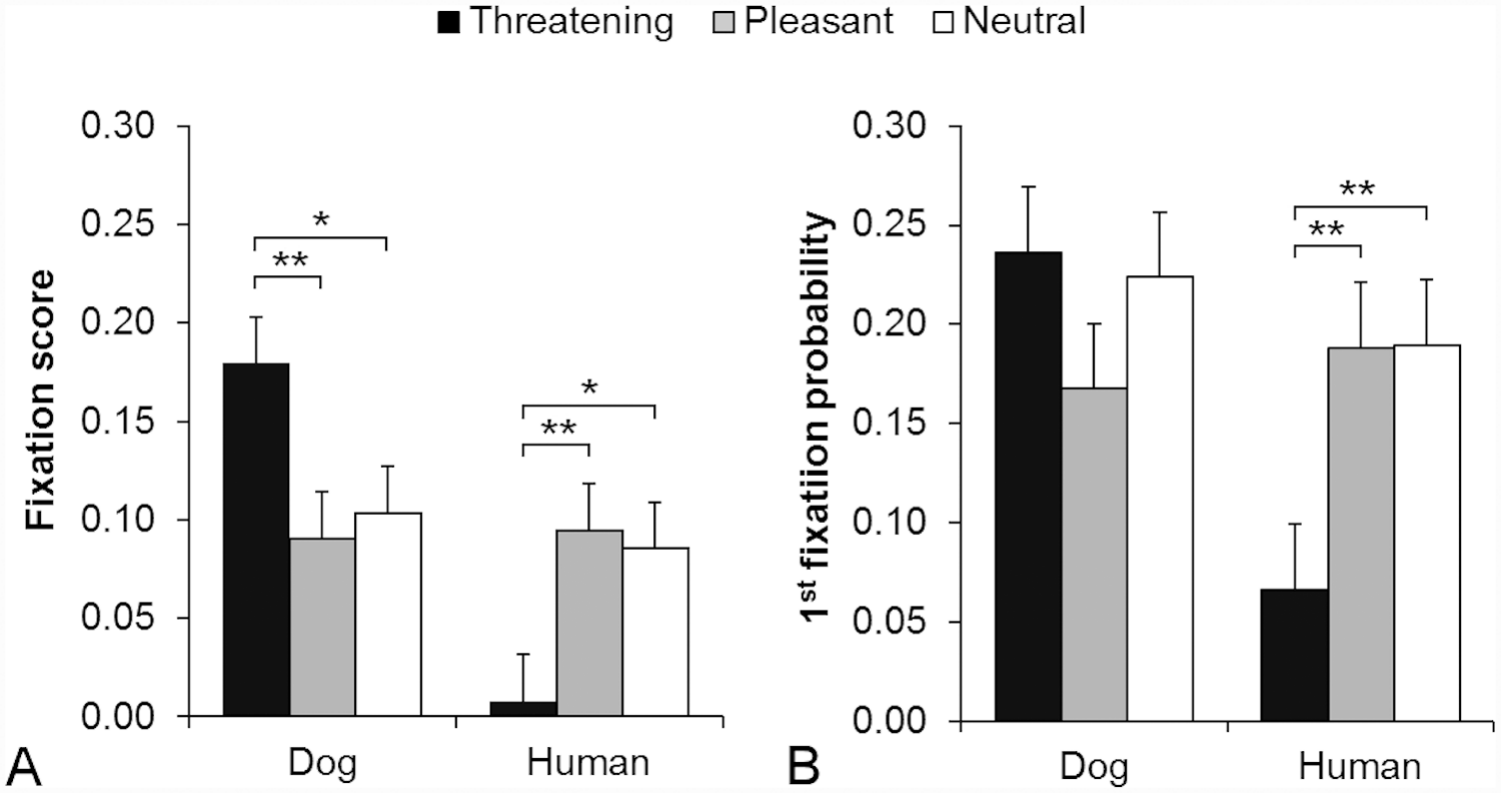
Expression dependent gazing behavior toward the inner face area. A) Fixation score B) 1^st^ fixation probability for inner face area (eyes, midface and mouth combined) during viewing different facial expressions (Threatening, Pleasant and Neutral) of dogs and humans. The brackets with asterisks indicate statistically significant differences among the expressions within the depicted species (** *p* ≤ 0.01, * *p* < 0.05).

The fixation scores toward the inner face area (i.e. the eyes, midface and mouth combined) did not differ significantly among the different facial expressions (main effect of the MIXED, *p* = 0.994). However, the interaction between the depicted species and expressions was statistically significant (*p* < 0.001). The post hoc tests revealed that dogs looked longer at the inner face of Threatening dogs compared with the inner face of Pleasant or Neutral dogs (Fig 2A), suggesting heightened attention toward the threatening signals of conspecifics. In contrast, when human faces were presented, the response was reversed (Fig 2A). Examples of the looking patterns of dogs are shown in Fig 1 and S2 Movie.

The fixation scores toward the inner face area differed significantly between the depicted species (main effect, *p* < 0.001; dog 0.124 ± 0.017, human 0.063 ± 0.017). Considering the significant interaction between the depicted species and expressions (*p* < 0.001), the post hoc tests revealed that dogs looked at inner faces of dogs longer compared with the inner faces of humans only in the Threatening expression (*p* < 0.001, Fig 2A).

#### 1^st^ fixation probability

The probability of the 1^st^ fixation to land on the inner face area did not differ significantly among the facial expressions (main effect, *p* = 0.173). However, the interaction between the depicted species and expressions was statistically significant (*p* = 0.003). The post hoc tests revealed that the inner faces of Threatening humans received lower likelihood to be the targets of the first fixations compared with the inner faces of Pleasant or Neutral humans (Fig 2B).

The probability of the 1^st^ fixation to land on the inner face area differed significantly between the depicted species (main effect, *p* = 0.008; dog 0.213 ± 0.023, human 0.149 ± 0.023). Considering the significant interaction between the depicted species and expressions (*p* = 0.003), the post hoc tests revealed that the inner faces of Threatening dogs received higher likelihood to be the targets of the first fixations compared with the inner faces of Threatening humans *(p* < 0.001, Fig 2B).

#### Entry time

The average entry time toward the inner face was 435.2 ms ± 22.0 ms with no statistically significant main effects for the expressions (*p* = 0.845) or the depicted species (*p* = 0.598). The interaction between the depicted species and expressions was not statistically significant (*p* = 0.893).

### Gazing behavior toward eyes, midface and mouth

#### Fixation score

**Fig 3:**
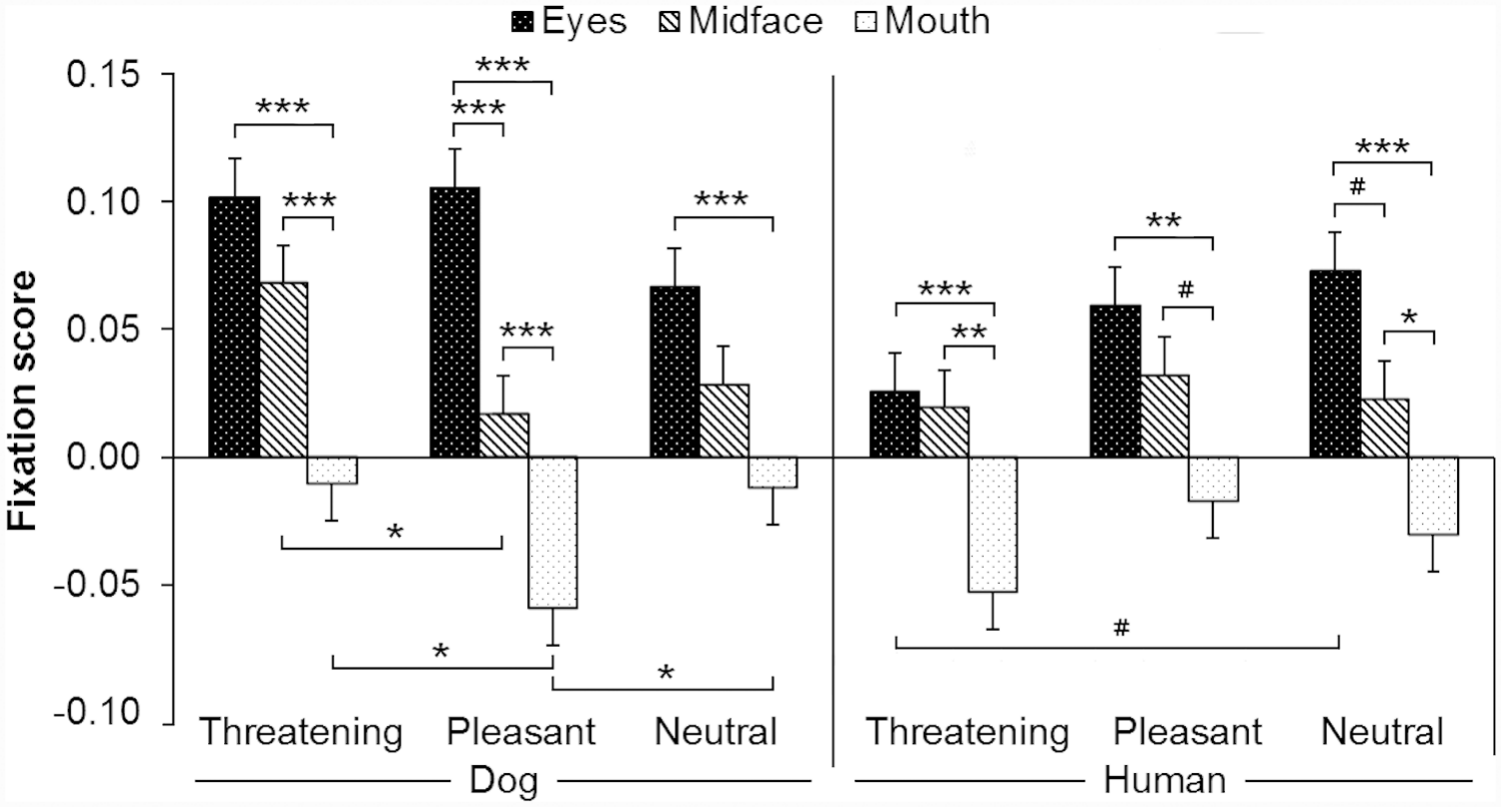
Expression dependent looking duration distribution among eyes, midface and mouth areas. The fixation scores for target facial features (eyes, midface and mouth) of the three facial expressions (Threatening, Pleasant and Neutral) of dogs and humans. The brackets above the bars indicate statistically significant differences among the target facial features within the species and the brackets below the bars differences among the expressions within the species (*** *p* ≤ 0.001, ** *p* ≤ 0.01, * *p* < 0.05, ^#^ *p* < 0.08).

The fixation scores toward different target facial features differed significantly from each other (main effect, *p* < 0.001; eyes 0.069 ± 0.007, midface 0.031 ± 0.007 mouth -0.031 ± 0.007). In addition, the interaction among the depicted species, expressions and target facial features was statistically significant (*p* < 0.001). The post hoc tests revealed that dogs looked longer at the eye area compared with the mouth area irrespective of the depicted species or expression (Fig 3).

The fixation scores toward the target facial features did not differ significantly among the expressions (main effect, *p* = 0.940). However, the interaction among the depicted species, expressions and target facial features was statistically significant (*p* < 0.001). The post hoc tests revealed that dogs looked longer at the mouths of Threatening and Neutral dogs compared with the mouths of Pleasant dogs (Fig 3). In addition, dogs looked longer at the midfaces of Threatening dogs compared with the midfaces of Pleasant dogs (Fig 3).

The fixation scores toward the target facial features differed significantly between the depicted species (main effect, *p* = 0.003; dog 0.032 ± 0.006, human 0.014 ± 0.006). In addition, the interaction among the depicted species, expressions and target facial features was statistically significant (*p* < 0.001). The post hoc tests revealed that dogs looked longer at threatening dogs compared with threatening humans within all facial feature areas (within the eyes *p* < 0.001; midface *p* = 0.019; mouth *p* = 0.027, Fig 3). Further, dogs looked at the eyes of Pleasant dogs longer than at the eyes of Pleasant humans (*p* = 0.032, Fig 3) and the mouths of Pleasant dogs shorter than the mouths of Pleasant humans (*p* = 0.030, Fig 3).

#### 1^st^ fixation probability

**Fig 4:**
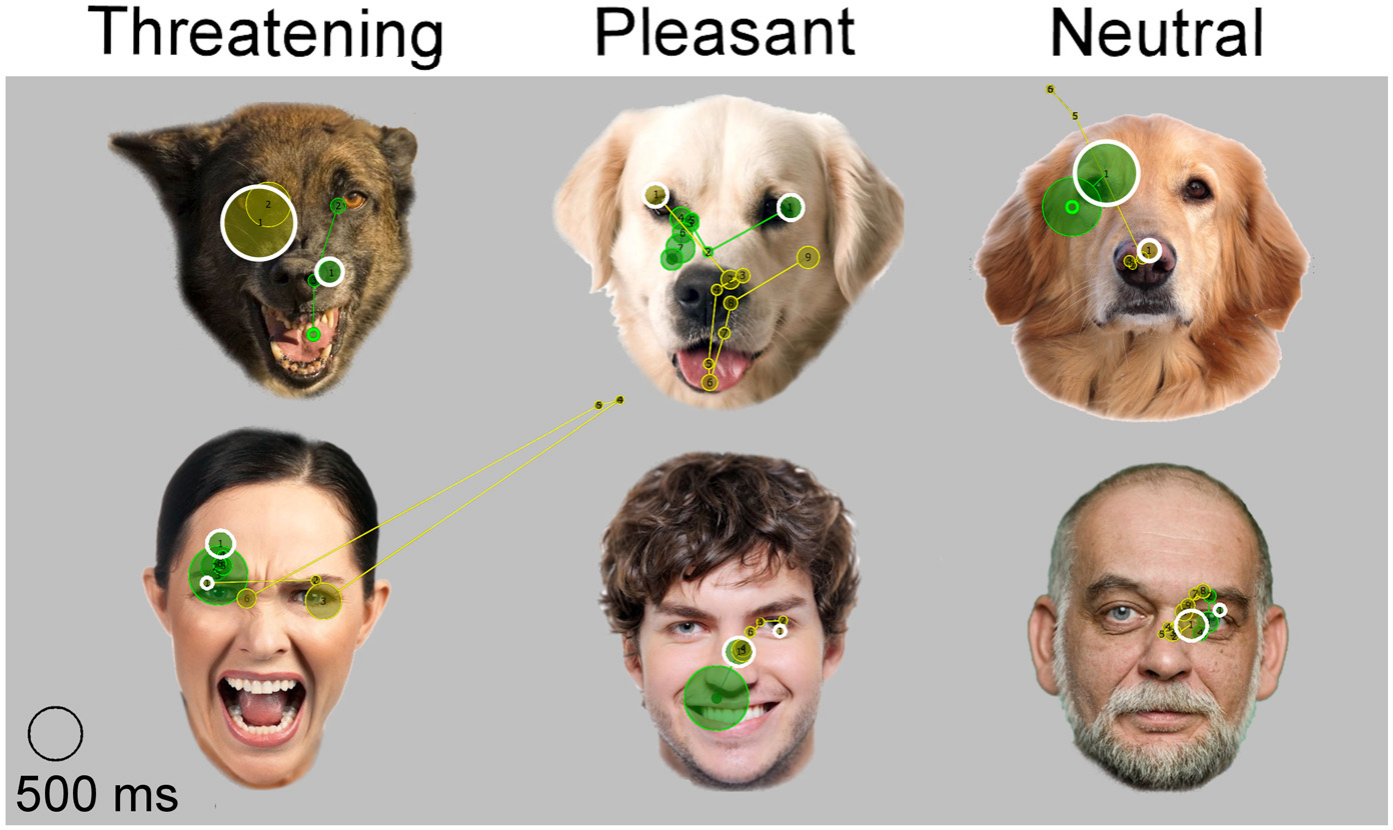
Examples of the first fixation targets and scanning paths. Fixations (circles) and scanning paths (lines between the circles) of two dogs during the 1500 ms presentations of three facial expressions (Threatening, Pleasant and Neutral) of dogs and humans faces. Different dogs are marked with different colors. White circles represent the targets of the first fixations. The original photos by S. Somppi and 123rf^®^. The images were purchased with a license to publish these images in electronic and printed documents and reports.

The 1^st^ fixation probabilities differed significantly among the target facial features (main effect, *p* < 0.001; eyes 0.092 ± 0.009, midface 0.056 ± 0.009, mouth -0.023 ± 0.009). The post hoc test revealed that eyes received higher likelihood to be targets of the first fixations compared with the midface (*p* = 0.016) or the mouth areas (*p* < 0.001) and the midface received higher likelihood compared with the mouth (*p* < 0.001). Furthermore, the depicted species differed significantly from each other in the 1^st^ fixation probabilities (main effect, *p* = 0.017; dog 0.051 ± 0.007, human 0.030 ± 0.008). The main effect for the expressions was not statistically significant (*p* = 0.312), neither was the interaction among the depicted species, expressions and target facial features (*p* = 0.069). Examples of the first fixation targets are shown in Fig 4.

#### Entry time

The gaze entry times to the different target facial features differed significantly (main effect, *p* = 0.002; eyes 503.4 ms ± 19.3 ms, midface 501.0 ms ± 20.3 ms, mouth 583.9 ms ± 24.2 ms). The post hoc test revealed that dogs fixated at the eyes and the midface areas faster than the mouth area (eyes vs. mouth *p* = 0.005 and midface vs. mouth *p* = 0.005). The main effects for the expression or the depicted species were not statistically significant (*p* = 0.576 and *p* = 0.628, respectively), neither was the interaction among the depicted species, expressions and target facial features (*p* = 0.966).

## Discussion

In this study, we broadened the scope of facial expression research to include the gazing behavior of domestic dogs (*Canis familiaris*). The present evidence confirms previous behavioral observations suggesting that dogs differentiate among depicted facial expressions [44–46] and adjust their viewing behavior spontaneously according to the observed emotion [46]. To our knowledge, this is the first evidence of emotion-related gaze patterns in non-primates. Despite primates and canids being distant in the phylogenetic tree, and their facial expressions having species-specific characteristics, the gazing behavior of dogs resembles the visual strategies of humans: their fixations are distributed among facial features systematically and their scanning behavior is modulated by facial expressions, especially that of threat. These phenomena may be based on phylogenetically old mechanisms.

Dogs scanned the facial features of conspecifics and humans in a similar manner from the initial attention: they fixated faster the eyes and midface areas than the mouth areas, and eyes received the higher probability to be the targets of the first fixations than the midfaces and the mouths. Likewise, humans, chimpanzees and macaque monkeys typically direct their first gaze preferentially on eyes [25,26,28,31,53,54] or just below them [23,31,55,56]. The initial focus is probably guided by a pre-attentive mechanism, which maximizes perceptual encoding performance [28,56].

Generally, dogs gazed at eyes overall longer than mouth areas regardless of the depicted species. Correspondingly, humans [24,26,28–31,57], chimpanzees [25,52,57], gorillas (*Gorilla gorilla*), orangutans (*Pongo abelii*) [57] and macaques [10,27,51,53,54] exhibit such fixation distribution among facial features (eyes > midface > mouth), even during viewing other species’ faces [10,25,31,53,57], suggesting that face-like configurations trigger systematic scanning behavior automatically [31]. The present results confirm the previous assumption that eyes play a significant role in the face perception of dogs [50]. In humans the intenseness to look at eyes may have developed to serve language-like functions, as joint attentional and communicative interactions, which requires collecting eye information actively [25]. Taking into account that domestic dogs resemble humans in their gaze communication (for reviews, see [34,35]), the analogous explanation may also apply to dogs.

Dogs’ gaze fixation distribution among the facial features was altered by the facial expression. In humans, expression-specific conspicuous visual properties (i.e. contrast, luminance, shape etc.) facilitate orienting toward particular facial structures [23]. However, physical saliency does not solely explain the locations of fixations [24], but attention is rather focused on the cues that are most relevant for encoding the certain emotion [24,26,28,29]. For example, humans view the mouths of pleasant faces most, probably because the mouth area is a key element for the detection and recognition of the human smile [23,24,29]. Dogs instead looked longer at the mouths of threatening and neutral dogs compared with the mouths of pleasant dogs. Likewise, chimpanzees [25] and rhesus monkeys [27] show heightened attention toward threatening mouths compared to pleasant mouths. For these animals the mouth area may be highly important for the interpretation of threatening expressions. Also the midface of threatening conspecifics’ seems to be salient for dogs. Instead macaques attend the midface area most in pleasant (lipsmack) monkey faces [27], and humans in disgust and sad human faces [29]. Although these expressions represent different emotions, they all include distinctive wrinkled muzzle/nose gestures, which may attract attention. The fixations of dogs could accumulate in midface area also because dogs might covertly observe the eyes of potentially threatening individuals by fixating near to eyes, but not directly on them. However, dogs did not actually avoid the eyes of threatening conspecifics. Instead, during exposure to human faces dogs tended to look at angry eyes less than neutral eyes, which could be due to the eye contact aversion, as in humans [18]. Overall, the features which are characteristic of species specific expressions appear to attract attention. Despite the variation in fixation distributions among the expressions, dogs keep the main focus on the eyes irrespective of the seen expression, corresponding to gazing behavior of humans [24,26,28–30].

The examination of the inner facial features as a whole revealed that the threatening faces evoked unique gazing patterns compared with the pleasant or neutral faces, referring to distinct visuo-cognitive processing strategies for threat-related facial expressions [11]. The result suggests that dogs do not base their perception of facial expressions on viewing of single structures, but the interpretation of the composition formed by eyes, midface and mouth. Dogs may process facial expressions holistically, at least threatening expressions. Further research is needed to clarify what perceptional strategy (purely visual saliency, featural or holistic [23,24,28,29]) dogs use in the recognition of facial expressions.

Notably, the attentional bias was dependent on the depicted species: dogs looked at the threatening dog faces for longer than at the other expressions of conspecifics, but scanned the threatening human faces in a contrary way, apparently avoiding looking at them. In primates including humans, threatening stimuli commonly enhance attention toward threatening conspecifics, which has been documented as accelerated initial orientation toward threat [8,9] or overall prolonged attention [9,11,12,16] which is the consequence of delay in disengaging the attention away from threat [13–15,58]. In dogs, we did not find evidence of faster orientation toward threatening faces: in terms of the entry time, dogs fixated on all expressions equally quickly. Instead, the sustained attention may arise from great interest toward threatening conspecific faces (interest guides dogs’ gaze fixations; [49,50]), or difficulties in disengaging the attention away from threat [13,58]. Such “stuck attention” has been suggested to be linked to a fear-induced freezing behavior, an evolutionarily important mechanism controlled by the amygdala [21,59]. The amygdala guides attention to emotionally significant stimuli, such as potentially harmful external events. The emotional response is further modulated by the prefrontal cortex, which enables more flexible behavioral outcomes. In arousing situations, the amygdala overrides prefrontal modulation and leads to failure to disengage attention away from the threatening stimuli (for reviews, see [21,22,59]). Arousal and fear relevance tend to be positively correlated with heightened attention [16,20,27,58]. Thus, it is possible that the images of aggressive dogs held attention because dogs find them more arousing than the images of angry humans. However, without physiological measurements we are not able to draw any conclusions about the subjective emotional state of dogs.

Dogs appeared to deem depicted angry human faces aversive, as has been proposed by the behavioral observations [45]. The avoidance of threatening stimuli is typical of anxious humans (for reviews, see [20,22]) but has also been observed in healthy humans [17,18] and monkeys during stressful emotional states [9]. Dogs avert their gaze from threatening human faces as early as the first fixations, which suggests that dogs can recognize the valence of the expression by their covert attention [60] even before the first fixations. Rapid gaze avoidance has been linked to submissive behavior [19]. Considering dogs’ flexible inter-species social communication [34,35] and their sensitivity to human threat signals [36,61], the gaze aversion from the threatening human faces could be a manifestation of appeasing behavior. This may be a learned mannerism, as in humans [18], or domestication may have equipped dogs with a propensity to display conflict-avoiding signals toward humans. In general, dogs show more common and severe aggressive reactions toward other dogs than people [62–63]. Selection for reduced human-directed aggression has likely shaped dogs to be highly tolerant to humans [64]. Flexible utilization of conflict-resolving behaviors may be a prominent element of domestic dogs’ social competence, which has enabled them to form peaceful mixed-species groups with humans [61,64].

Differential scanning of own-species’ and other-species’ threat signals may arise also from biological relevance of the stimuli [15,65,66]. Dog and human faces are both social and likely also emotional stimuli for dogs, but faces of conspecifics may carry more biological significance for them. Biological emotional stimuli (e.g. object relevant to survival or reproduction) and social emotional stimuli (e.g. object relevant to social life) are perhaps processed in different pathways and thus they alter attention contrarily [65–67]. In humans, biologically relevant images are detected rapidly and they facilitate attention engagement because of the strong amygdala activity and interconnection between the amygdala and the visual cortex. Instead, socially emotional images evoke greater activity in the prefrontal cortex-based circuit [59,65,67]. We propose that in the present study the initial attention was driven by the earlier limbic pathways during the viewing of both canine and human faces. Further, the biologically more relevant threatening conspecific faces were mainly processed via this route, which led to heightened attention. Threatening human faces received more complex prefrontal processing instead, which led to the avoidance response. The enhanced prefrontal modulation could be linked to submissive behavior [19] and/or memory-based social evaluations [66].

The subjects’ current emotional state (e.g. stress) and personality (e.g. anxiousness and neuroticism) [9,20,22,59], and also the stimulus types and paradigm may have affected the results in our study. In future studies the interaction among phylogenetic, biological and personal validity of the stimuli should be taken into account. For example, do dogs exhibit attentional bias toward biological stimuli that evoke a phylogenetic fear response (e.g. snakes) versus non-biological stimuli, which evoke an ontogenetic fear response (e.g. nail clippers; for examples of paradigms, see [15,58,65–67])? The physical saliency of the stimuli should be controlled by using saliency models [68] and stimuli that are not socially relevant, but morphologically similar to the dog faces (e.g. other animal faces or artificial stimuli). In addition, the investigation should be broadened to other emotionally informative facial structures, including their movements, for example ears [27,42].

In conclusion, during viewing emotional faces of conspecifics and humans, domestic dogs’ gaze fixations spread systematically among facial features: regardless of the viewed expression, eyes were the most probable targets of the first fixations and gathered overall more attention than mouth area. The primacy of eyes suggests that eyes are physically salient and/or highly informative facial feature for dogs. However, dogs did not base their perception of facial expressions on viewing single facial structures, but the interpretation of the composition formed by eyes, midface and mouth. They evaluated social threat signals rapidly, and this evaluation led to attentional bias, which emerged as enhanced attention or avoidance, depending on the biological validity of the stimuli. We propose that the biologically more relevant threatening conspecific faces are processed mainly by the earlier limbic pathways, which results in enhanced attention. In contrast, the avoidance response induced by the threatening signals of humans may be more modulated by the prefrontal cortex. These two mechanisms may both have an adaptive significance for domestic dogs. The tolerant behavior strategy of domestic dogs toward humans may partially explain the results. The present findings bring a new perspective to understanding the processing of emotional expressions in non-primate animals. Exploring further the sensitivity to social threat in canids provides a novel comparative approach to unravel the etiology of neurocognitive-affective disorders that exist in both humans and dogs, such as social phobias and anxiety.

## Supporting Information

S1 Dataset The data file including raw gaze variables. One sample represents the gazing behavior during one stimulus image presentation.
(XLSX)

S2 Dataset The data file including the averaged gaze variables. One sample represents the mean gazing behavior averaged over all image presentations of one stimulus category (per a dog, per a day).
(XLSX)

S1 Movie An example of a measurement session demonstrating how dogs settled down in the pre-trained position and viewed a block of stimuli while their eye gaze was recorded with a contact-free eye-tracker. The original photos used as stimuli images by A. Pikkusaari, MS ClipArt, 123rf^®^ and BigStock. The images were purchased with a license to publish these images in electronic and printed documents and reports.
(MP4)

S2 Movie An example of a stimulus block with the visualized scanning patterns of dogs. The original photos by S. Somppi and 123rf^®^. The images were purchased with a license to publish these images in electronic and printed documents and reports.
(MP4)
